# Sirolimus treatment of severe congenital hyperinsulinism

**DOI:** 10.1186/1687-9856-2015-S1-P123

**Published:** 2015-04-28

**Authors:** Lisa A Amato, Charmian A Quigley, Kris A Neville, Shihab Hameed, Charles F Verge, Helen J Woodhead, Jan L Walker

**Affiliations:** 1 Endocrinology, Sydney Children's Hospital, Sydney, NSW, Australia; 2 School of Women’s and Children’s Health, UNSW, Sydney, NSW, Australia

## 

Sirolimus treatment reduced dependence on octreotide and frequent feeding in 4 infants with CHI in a recent study [1]. We report our experience in a 1 year-old boy with presumed diffuse disease due to a de novo heterozygous ABCC8 missense mutation (p.D1506E).

This macrosomic infant (term birth weight 5.676kg) presented with hypoglycaemia in the first hours of life. Critical sample results were consistent with CHI (blood glucose 0.9mmol/L, insulin 41mU/L, free fatty acids 0.2mmol/L[RR 0.1-0.6]). The family history was significant for type 2 diabetes in both grandmothers, and CHI in his maternal second cousin. No ABCC8 missense mutation was identified in either parent or his second cousin.

Initial management included intravenous glucose (12mg/kg/min) and glucagon infusions. Maximal dose diazoxide (20mg/kg/day for 5 days) was ineffective so subcutaneous octreotide infusion was initiated (26mcg/kg/day). At discharge from hospital (age 10 weeks) he was overweight (height SDS 1.11, weight SDS 3.1), receiving octreotide 27mcg/kg/day, nocturnal gastrostomy Polyjoule® feeds (equivalent to 11mg/kg/min glucose) plus 3-hourly daytime bolus feeds.

Sirolimus (0.5mg/m2/day) was added at age 7 months because of continued weight gain and intermittent mild hypoglycaemia while receiving octreotide 37mcg/kg/day. The dose was increased to 6mg/m2/day, aiming for trough levels 15-20ng/ml. After 3 months, the octreotide dose has stabilised (36mcg/kg/day) and the intensity of feeds has been reduced (3.5mg/kg/minute glucose overnight), resulting in weight loss; however, he has also developed linear growth failure (height velocity 6cm/year over 3 months). At 1 year of age his length and weight SDS are 0.48 and 1.21 respectively, and he is developmentally appropriate for age. Apart from one mild episode of respiratory syncytial virus, he has had no significant illnesses during sirolimus treatment.

In conclusion, treatment with sirolimus has improved our patient’s blood glucose concentrations, decreased his dependence on hypercaloric nocturnal continuous feeds and decreased his weight gain; however the burden of care remains more significant than reported in the 4 published cases, 2 of whom had heterozygous mutations in ABCC8. The combination of high-dose octreotide and sirolimus may not be sustainable long-term in view of their likely combined contribution to his growth failure and the potential for immunosuppression due to sirolimus.

Written informed consent was obtained from the patient's parent or guardian for publication of this Case report (and any accompanying images). A copy of the written consent is available for review by the Editor-in-Chief of this journal.
